# Site‐specific trends in gastroenteropancreatic neuroendocrine neoplasms in Bavaria, Germany

**DOI:** 10.1002/cam4.6510

**Published:** 2023-09-22

**Authors:** Nina Grundmann, Sven Voigtländer, Amir Hakimhashemi, Ulrich‐Frank Pape, Martin Meyer, Jacqueline Müller‐Nordhorn

**Affiliations:** ^1^ Bavarian Cancer Registry, Bavarian Health and Food Safety Authority Nuremberg Germany; ^2^ Department of Internal Medicine and Gastroenterology, Asklepios Tumour Centre Hamburg and Asklepios Hospital St. Georg Hamburg Germany

**Keywords:** gastroenteropancreatic neuroendocrine tumor, gastrointestinal neoplasms, incidence, neuroendocrine carcinoma, neuroendocrine tumors

## Abstract

**Introduction:**

Neuroendocrine neoplasms (NEN) are rare and heterogeneous epithelial tumors, occurring throughout the body. For gastroenteropancreatic (GEP)‐NEN, rising incidence rates were reported for the last decades, with underlying causes remaining largely unexplained. We evaluated NEN trends stratifying by their histologic subtypes.

**Methods:**

Incident cases of GEP‐NEN diagnosed between 2005 and 2019 were retrieved from the prospective, population‐based Bavarian Cancer Registry. GEP‐NEN were divided in their histologic subtypes, that is, neuroendocrine tumors (NET) G1, NET G2/G3, other NET versus small‐cell neuroendocrine carcinoma (NEC), large‐cell NEC, and other NEC. We calculated annual age‐standardized incidence rates (ASIRs) per 100,000 persons for the total of GEP‐NEN, NEN histologic subtypes, and tumor sites. We used an annual percentage change (APC) approach including a joinpoint analysis to investigate NEN incidence trends.

**Results:**

ASIR of GEP‐NEN rose from 2.2 in 2005 to 4.8 in 2019, characterized by a significant increase until 2012 (APC 2005–2012: 10.1%), followed by modest rise (APC 2012–2019: 1.5%). In the last decade, this increase was mainly driven by the rise of NET G1 and G2/G3, while incidence for NEC declined. Over the study period, ASIR increased significantly for all GEP‐sites except the colon. APCs were largest for the stomach, the appendix, the pancreas, and the rectum.

**Conclusions:**

This study found a significant increase in the incidence of GEP‐NET. Though this development may partially be attributable to the increased use of advanced detection techniques and changes in NEN classification, further research should also focus on the identification of NEN risk factors.

## Graphical abstract

Incidence trends of gastroenteropancreatic neuroendocrine neoplasms (GEP‐NEN) between 2005 and 2019 were analyzed, stratified by cancer site and by histologic subtype. Findings indicated a significant increase in age‐standardized incidence rates for all GEP‐sites with exception of the colon. The increase was mainly driven by the rise of neuroendocrine tumors, while neuroendocrine carcinoma declined.

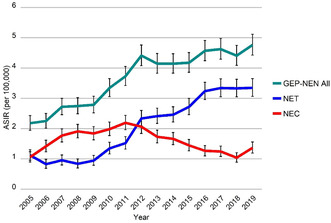

## 1 INTRODUCTION

Neuroendocrine neoplasms (NEN) are a rare and strongly heterogeneous group of tumors showing variable clinicopathologic and biologic behavior.^1^ They originate from neuroendocrine cells,^2^ which express the vesicle‐characteristic proteins synaptophysin and chromogranin A,^3^ hallmark neuroendocrine markers used for entity‐specific immunohistochemical diagnosis as well as for the monitoring of disease progression. The majority of NEN is slow growing and indolent for a long period. This may lead to a delay in diagnosis and presentation of patients in a metastatic stage of the tumor, when a curative treatment is not possible anymore.^4^, ^5^ Symptoms vary according to the tumor site.^1^ Localization, histology, and tumor size are the best predictors for disease development and serve as the basis for treatment decision.^6^ Following the distribution of neuroendocrine cells, NEN can occur throughout the body. Approximately 65% of all NEN are localized in the gastroenteropancreatic (GEP) system, about 25% in the lung and the bronchi (excluding small cell neuroendocrine carcinoma), and about 10% in other sites, for example, the thymus or the ovaries.^1^ In this study, we focus on NEN located in the GEP system.

The European Neuroendocrine Tumour Society (ENETS) issued standard of care guidelines for pathology in 2009. Since then, profound changes in the classification have been made. The ENETS proposed a unified staging system with the aim of facilitating the assessment and stratified management of NEN according to behavior and prognosis.^7^, ^8^, ^9^ After initial validation studies, NEN were divided for the first time into neuroendocrine tumors (NET) and neuroendocrine carcinoma (NEC) in the 4th edition of the World Health Organization (WHO) classification of tumors 2010.^10^ The latest classification of GEP‐NEN was published in the 2019 WHO classification of tumors (5th edition).^11^ It differentiated GEP‐NEN according to their proliferation characteristics into well‐differentiated NET, graded Grade 1 (G1)–Grade 3 (G3), poorly differentiated NEC, and mixed neuroendocrine–non‐neuroendocrine tumors (MiNEN). The 7th edition of the TNM classification of malignant tumors of 2009^12^ included NEN for the first time, widely adapting ENETS proposals, though some differences regarding NEC as well as pancreatic and appendiceal NET persisted.^13^ The growing debate on NEN gave rise to further, continuous changes in the classification of NEN. Beyond other, these consisted of a change in consent on malignancy of specific histologic codes (‘behavior code’ change) and the inclusion of new histologic codes, for example, the functioning pancreatic neuroendocrine tumors (PanNETs), finally implemented in the first revision of the International Classification of Diseases for Oncology (ICD‐O3).^14^

Previous epidemiological research on GEP‐NEN reported continuously rising incidence rates for the last decades,^6^, ^15^, ^16^, ^17^, ^18^ predominantly for countries of Western Europe, North America, and from Japan. The United States, to give just one example, experienced a strong increase of GEP‐NEN rates, which rose 3.6‐fold between 1973 and 2007.^15^ Uncertainty exists whether the observed increase in NEN incidence is due to improved detection strategies of these tumors, altering risk factors, or changes in tumor biology. Published data from Germany and other European countries have often focused on retrospective analysis of oncological studies,^19^ mostly obtained from referral centers, or the analysis of specific subgroups.^20^, ^21^ Due to national restrictions and data protection rules, large population‐based data such as provided by the Surveillance, Epidemiology, and End Results (SEER) Program in the United States^5^, ^22^ are often not available for Europe. In particular, regarding the more recent changes in the WHO and TNM classifications, population‐based incidence data are needed.

This study aims to analyze time trends in the incidence of GEP‐NEN in a population‐based cancer registry between 2005 and 2019 in Bavaria, Germany, stratified by age, NEN subtype, tumor site, and stage. Analyzing time trends in subgroups can contribute to the exploration of the increase in NEN incidence observed in many countries.

## 2 METHODS

Data were retrieved from the population‐based Bavarian Cancer Registry, which at present covers a population of more than 13 million residents. Being established in 1998, it records all cancer cases in Bavaria reported by inpatient and outpatient facilities.^23^ We included all incident cases of malignant NEN, diagnosed between 2005 and 2019, as for most GEP entities, completeness of coverage was achieved for these years. Completeness of coverage, which is estimated by the ratio of mortality to incidence, is a data quality measure of cancer registries.^24^ Internationally, it serves as an indicator of data reliability in cancer registry‐based research. The percentage of death certificate only (DCO) cases continuously declined, from 2009 onwards falling below 15%.

We identified malignant GEP‐NEN by their morphologic codes as well as behavior code (code 3: malignant), based on the 2019 WHO classification of tumors of the digestive system (5th edition), also known as the Blue Books.^11^ Benign tumors were not considered because they have not been systematically registered in the Bavarian Cancer Registry. GEP‐NEN sites were defined by the topographical codes C15–C25 based on the International Classification of Diseases, Tenth Revision (ICD‐10). Cases of GEP‐NEN were further differentiated into NET (G1, Grade 2/3 [G2/G3], other NET), NEC (small‐cell NEC [SCNEC], large‐cell NEC [LCNEC], other NEC) and MiNEN according to the morphologic codes of the latest WHO classification.^11^ Small‐cell (SC, morphologic code 8041/3) and large‐cell (LC, 8013/3) neuroendocrine carcinoma were classified as SCNEC and LCNEC, respectively, and NEC, not otherwise specified (NOS) (8246/3) as other NEC. Carcinoid tumors NOS (8240/3) were classified as NET G1 and atypical carcinoid tumors (8249/3) as NET G2/3; mixed neuroendocrine–non‐neuroendocrine neoplasm (8154/3) were classified as MiNEN, including mixed adenoneuroendocrine carcinoma (8244/3), only for esophageal and gastric NEN. Non‐functioning (NF‐PanNETs, 8150/3) and functioning pancreatic neuroendocrine tumors (PanNETs, i.e., insulinoma (8151/3), glucagonoma (8152/3), gastrinoma (8153/3), vipoma (8155/3), somatostaninoma (8156/3), ACTH‐producing NET (8158/3), and serotonin‐producing NET (8241/3), as well as enterochromaffin‐like cell tumors (8242/3, only for the stomach) and extra‐adrenal paraganglioma (8693/3, only for the small intestine) were classified as other NET. In principle, 8249/3 comprises NET G2 as well as NET G3, which differ regarding their proliferation behavior (mitotic counts or Ki67‐based proliferative index below/equal vs. above 20). However, a differentiation of NET G2 and NET G3 was not possible due to a lack of additional information apart from the morphologic code. Thus, NET G2 and NET G3 had to be considered as one category (NET G2/G3). For the appendix, we additionally accounted for the behavior code change of the histology 8240 from behavior code 1 (“borderline malignancy”) to behavior code 3 (“malignant”) with the first revision of ICD‐O3 in 2013^14^ to avoid misclassification.

Annual age‐standardized incidence rates (ASIRs) per 100,000 persons with corresponding 95% confidence intervals (CI)^25^ were calculated for the total of GEP‐NEN and its histologic subtype (NET G1, NET G2/G3, other NET, SCNEC, LCNEC, and other NEC), as well as for each GEP site, using the European standard population^26^ and the average Bavarian population of the respective year. To evaluate GEP‐NEN time trends, we applied an annual percent change approach (APC) including joinpoint analysis and also calculated average APCs (AAPCs) for the whole study period 2005–2019.^27^ APCs as well as AAPCs were estimated with corresponding 95% CI for all GEP‐NEN, their histologic subtypes, GEP sites, and stages, according to Union for International Cancer Control (UICC). We used a tiebreak method^28^ by adding the minimum value for the ASIR in the time series to each component to approximate the APCs and AAPCs of NET G2/G3. Specific sites were selected for further analysis, including stratification by age group, pre‐defined in 0–39, 40–59, 60–79, 80+ years of age, and UICC stage. The statistical analyses were performed using SAS (SAS Institute, Inc.), version 9.4 and R (R Foundation for Statistical Computing), version 4.1.1.

## 3 RESULTS

Between 2005 and 2019, a total of 9236 cases of GEP‐NEN were identified from the registry data (Table 1). The most frequent GEP‐NEN site was the small intestine (30.8%), followed by the pancreas (19.8%). NEN were slightly more prevalent in men (54.0%) than in women (46.0%), with the exception of the appendix with almost two thirds (61.0%) occurring in females. Patients presented at first diagnosis with an average age of 61 years (±16.2 years) of age. Mean age was lowest for the appendix (43 ± 21.4 years). The largest proportion of GEP‐NEN was diagnosed in UICC Stage IV (21.2%). A high percentage of cases had no staging (UICC Stage X: 44.5%). For most sites, NEN cases increased with age. However, regarding appendix, almost half of the patients were <40 years old (48.5%). Within this age group, 66.8% of appendiceal NEN occurred in women.

**TABLE 1:** GEP‐NEN by site: Descriptive analysis of cases in Bavaria.

	Esophagus	Stomach	Small intestine	Colon	Appendix	Rectum	Anus	Bile/ Liver	Pancreas	Sum
*N* (%)	114 (1.2)	1249 (13.5)	2843 (30.8)	674 (7.3)	1106 (12.0)	1160 (12.6)	22 (0.2)	244 (2.6)	1824 (19.8)	9236 (100.0)
Sex^a^										
Male	79 (69.3)	658 (52.7)	1635 (57.5)	378 (56.1)	436 (39.4)	677 (58.4)	12 (54.6)	128 (52.5)	983 (53.9)	4986 (54.0)
Female	35 (30.7)	591 (47.3)	1207 (42.5)	296 (43.9)	670 (60.6)	482 (41.6)	10 (45.5)	116 (47.5)	840 (46.1)	4247 (46.0)
Age (years)										
0–39	2 (1.8)	65 (5.2)	81 (2.9)	24 (3.6)	536 (48.5)	119 (10.3)	3 (13.6)	7 (2.9)	109 (6.0)	946 (10.2)
40–59	34 (29.8)	343 (27.5)	834 (29.3)	171 (25.4)	295 (26.7)	465 (40.1)	6 (27.3)	56 (23.0)	594 (32.6)	2798 (30.3)
60–79	65 (57.0)	663 (53.1)	1553 (54.6)	369 (54.8)	210 (19.0)	495 (42.7)	10 (45.5)	144 (59.0)	988 (54.2)	4497 (48.7)
80+	13 (11.4)	178 (14.3)	375 (13.2)	110 (16.3)	65 (5.9)	81 (7.0)	3 (13.6)	37 (15.2)	133 (7.3)	995 (10.8)
Mean ± SD	64.8 ± 11.9	65.1 ± 13.7	65.3 ± 12.7	66.8 ± 13.5	43.0 ± 21.4	58.9 ± 14.4	61.5 ± 17.5	67.2 ± 12.8	62.5 ± 13.5	61.4 ± 16.2
UICC										
I	2 (1.8)	166 (13.3)	228 (8.0)	39 (5.8)	284 (25.7)	201 (17.3)	2 (9.1)	20 (8.2)	381 (20.9)	1323 (14.3)
II	5 (4.4)	58 (4.6)	163 (5.7)	49 (7.3)	107 (9.7)	10 (0.9)	1 (4.6)	17 (7.0)	291 (16.0)	701 (7.6)
III	19 (16.7)	35 (2.8)	764 (26.9)	168 (24.9)	55 (5.0)	58 (5.0)	1 (4.6)	15 (6.2)	33 (1.8)	1148 (12.4)
IV	44 (38.6)	149 (11.9)	641 (22.6)	248 (36.8)	26 (2.4)	149 (12.8)	5 (22.7)	34 (13.9)	658 (36.1)	1954 (21.2)
X	44 (38.6)	841 (67.3)	1047 (36.8)	170 (25.2)	634 (57.3)	742 (64.0)	13 (59.1)	158 (64.8)	461 (25.3)	4110 (44.5)
Previous tumor										
Yes	23 (20.2)	181 (14.5)	479 (16.8)	83 (12.3)	109 (9.9)	108 (9.3)	2 (9.1)	46 (18.9)	266 (14.6)	1297 (14.0)
GEP grading										
NET G1	3 (2.6)	583 (46.7)	1215 (42.7)	174 (25.8)	793 (71.7)	662 (57.1)	10 (45.5)	42 (17.2)	429 (23.5)	3911 (42.4)
NET G2/G3	0	128 (10.3)	356 (12.5)	40 (5.9)	24 (2.2)	55 (4.7)	1 (4.6)	23 (9.4)	291 (16.0)	918 (9.9)
Other NET	0	5 (0.4)	14 (0.5)	2 (0.3)	5 (0.5)	0	0	0	196 (10.8)	222 (2.4)
SCNEC	45 (39.5)	33 (2.6)	7 (0.3)	28 (4.2)	1 (0.1)	24 (2.1)	3 (13.6)	37 (15.1)	54 (3.0)	232 (2.5)
LCNEC	8 (7.0)	29 (2.3)	10 (0.4)	51 (7.6)	4 (0.4)	22 (1.9)	3 (13.6)	11 (4.5)	30 (1.6)	168 (1.8)
Other NEC	46 (40.4)	444 (35.6)	1241 (43.7)	379 (56.2)	279 (25.2)	397 (34.2)	5 (22.7)	130 (53.3)	805 (44.1)	3726 (40.3)
MiNEN	12 (10.5)	27 (2.2)	0	0	0	0	0	1 (0.4)	19 (1.0)	59 (0.6)

Abbreviations: G, grade; GEP‐NEN, gastroenteropancreatic neuroendocrine neoplasm; LC, large cell; MiNEN, mixed neuroendocrine–non‐neuroendocrine neoplasm; NEC, neuroendocrine carcinoma; NET, neuroendocrine tumor; SC, small cell; UICC, Union for International Cancer Control, classification of tumor stages.

^a^ Including three cases of unknown sex.

**FIGURE 1:**
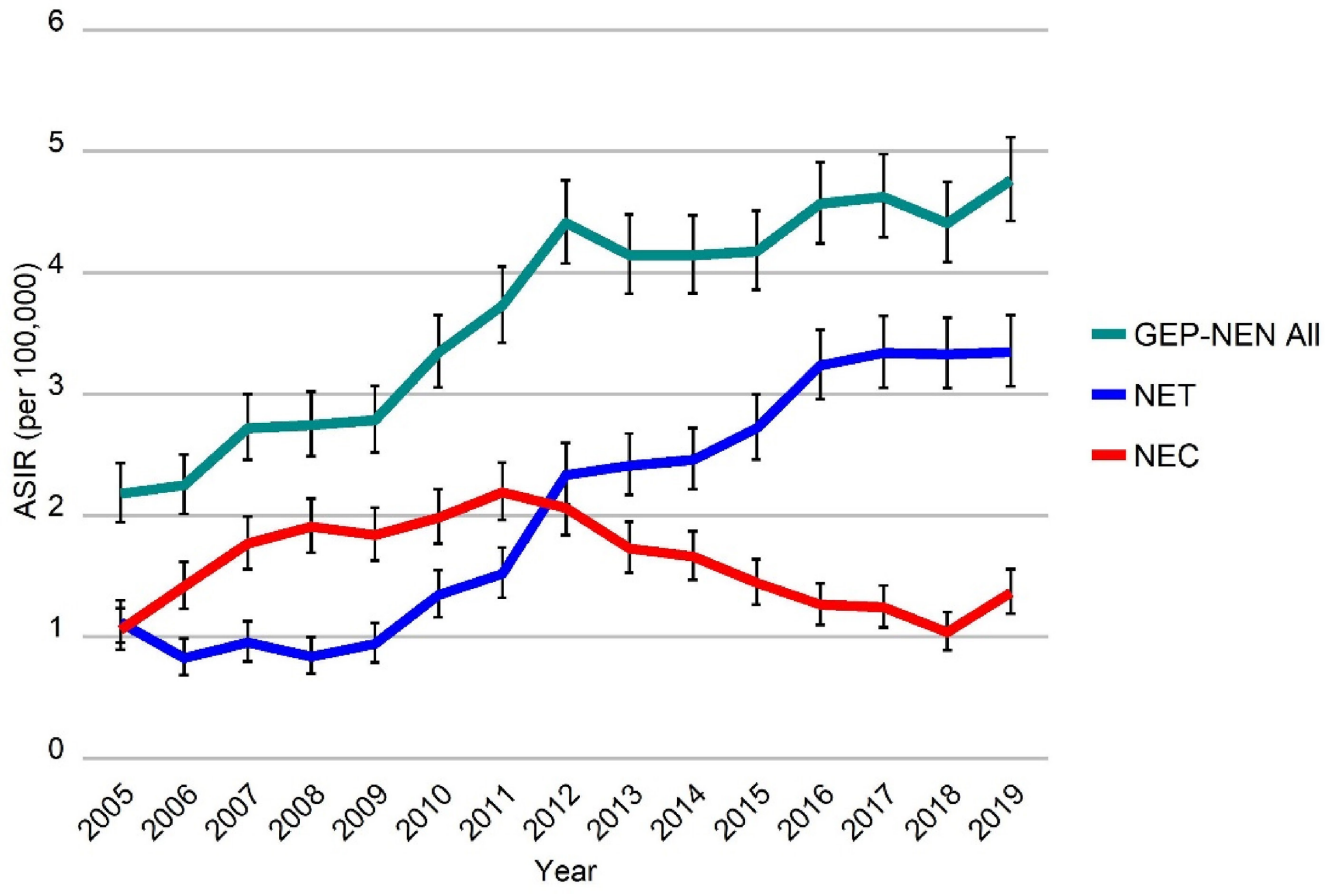
Age‐standardized incidence of GEP‐NEN, stratified by type of NEN. ASIR, age‐standardized incidence rate (European standard population); GEP‐NEN, gastroenteropancreatic neuroendocrine neoplasm; NEC, neuroendocrine carcinoma; NET, neuroendocrine tumor. For relevant data see Table S2.

**FIGURE 2:**
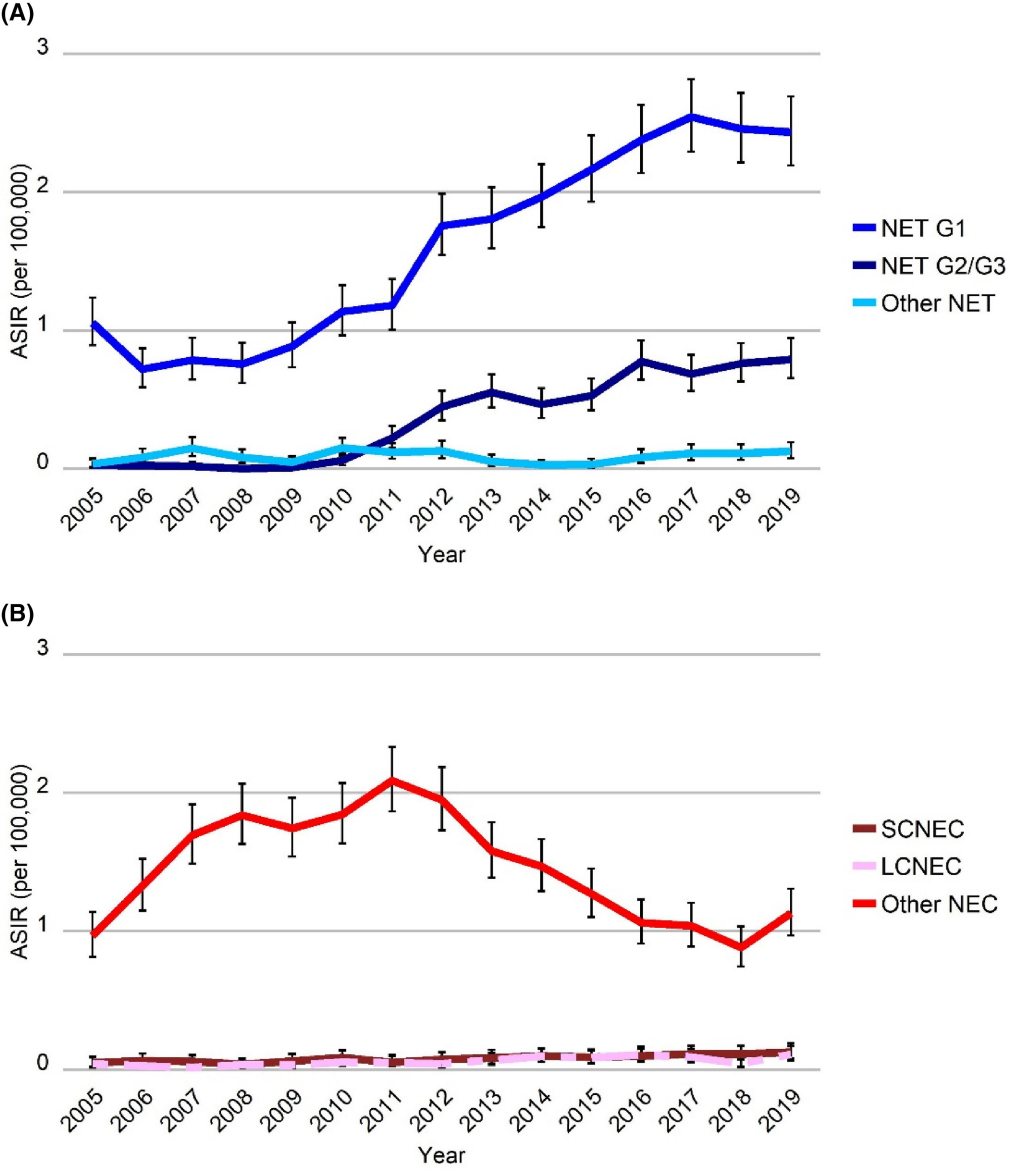
Age‐standardized incidence of (A) NET and (B) NEC, both stratified by histologic subtype. ASIR, age‐standardized incidence rate (European standard population); G, Grade; LC, large cell; NEC, neuroendocrine carcinoma; NET, neuroendocrine tumor; SC, small cell. For relevant data see Table S3.

Between 2005 and 2019, ASIR for all GEP‐NEN significantly increased from 2.2 per 100,000 residents in 2005 to 4.8 in 2019 (Table 2, Figure 1). Rates distinctly rose between 2005 and 2012 (APC 10.1% [95% CI 7.7%; 12.6%]), slowing down to an APC of 1.5% [95% CI 0.0%; 3.1%] from 2012 to 2019. Trends differed between the histologic subtypes of NET and NEC. NET incidence significantly increased between 2005 and 2019. Following an initial decrease (APC −13.7% [95% CI −51.9%; 54.8%]), NET G1 incidence increased significantly from 2007 onwards (APC 11.9% [95% CI 9.7%; 14.0%]) (Figure 2A). Incidence of NET G2/G3 increased from 0 in 2008 to 0.8 in 2019 (APC 46.2% [95% CI 24.4%; 71.8%]). Incidence of NEC, on the other hand, increased between 2005 and 2010 (APC 12.1% [95% CI 3.8%; 21.1%]) but subsequently decreased until the end of the study period (APC −7.1% [95% CI −9.7%; −4.5%]), mainly driven by unspecified NEC (other NEC) (Figure 2B). Incidence of SCNEC, LCNEC, and other NET remained rather stable. The inverse trends of NET and NEC were reflected in the analyses of GEP sites, stratifying NEN cases by histologic subtype (Figure S1).

**TABLE 2:** Trends in incidence of GEP‐NEN by histologic subtype.

Subtype	Period	ASIR baseline	ASIR end	AAPC (lower, upper CI 95%)	APC (lower, upper CI 95%) Period before/after joinpoint	*p*‐value
GEP‐NEN (all)	2005–2019	2.18	4.76	5.9 (4.5; 7.2)^b^		<0.001
	2005–2012	2.18	4.41		10.1 (7.7; 12.6)^b^	<0.001
	2012–2019	4.41	4.76		1.5 (0.0; 3.1)	0.059
SCNEC	2005–2019	0.05	0.13	7.1 (4. 5; 9.7)^b^		<0.001
LCNEC	2005–2019	0.04	0.11	10.6 (5.4; 16.0)^b^		<0.001
Other NEC	2005–2019	0.97	1.13	−2.7 (−5.9; 0.6)		0.102
	2005–2010	0.97	1.84		12.5 (3.0; 23.0)^b^	0.027
	2010–2019	1.84	1.13		−8.7 (−11.4; −5.8)^b^	<0.001
NET G1	2005–2019	1.06	2.43	10.5 (8.0; 13.0)^b^		<0.001
	2005‐2007	1.06	0.79		−13.7 (−51.9; 54.8)	0.475
	2007–2019	0.79	2.43		11.9 (9.7; 14.0)^b^	<0.001
NET G2/G3^a^	2005–2019	0.03	0.79	38.5 (24.7; 53.9)^b^		<0.001
	2005–2008	0.03	0.00		−37.2 (−65.3; 13.8)	0.130
	2008–2019	0.00	0.79		46.2 (24.4; 71.8)^b^	<0.001
Other NET	2005–2019	0.04	0.13	1.3 (−6.1; 9.3)		0.722
MiNEN	2005–2019	0.01	0.05	17.1 (9.6; 25.2)^b^		<0.001

Abbreviations: (A)APC, (average) annual percent change; ASIR, age‐standardized incidence rate per 100,000 residents (European standard population); CI, confidence interval; GEP‐NEN, gastroenteropancreatic neuroendocrine neoplasms; LCNEC, large cell NEC; MiNEN, mixed neuroendocrine‐non‐neuroendocrine neoplasms; NEC, neuroendocrine carcinoma; NET, neuroendocrine tumor; SCNEC, small cell NEC.

^a^ Use of tiebreak method due to ASIR (2008) = 0.

^b^ Indicates that APC is significantly different from zero based on a significance level of 5%.

**FIGURE 3:**
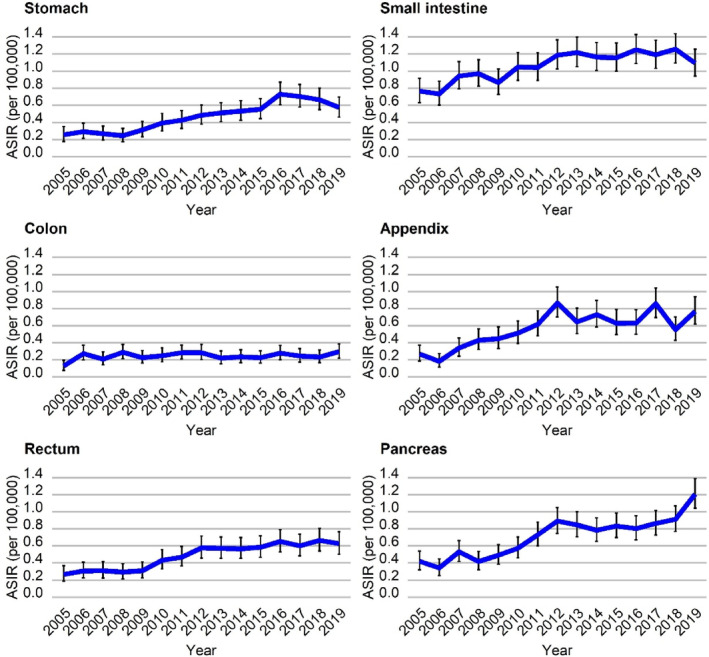
Age‐standardized incidence of GEP‐NEN, stratified by tumor site: stomach, small intestine, colon, appendix, rectum, and pancreas. ASIR, age‐standardized incidence rate (European standard population); GEP‐NEN, gastroenteropancreatic neuroendocrine neoplasm. For relevant data see Table S4.

Between 2005 and 2019, NEN ASIR increased for all GEP sites (Figure 3). The highest increase was found for the stomach (AAPC 8.3% [95% CI 6.4%; 10.2%]), followed by the appendix (AAPC 8.1% [95% CI 4.4%; 12.0%]), the pancreas (AAPC 7.7% [95% CI 5.6%; 9.7%]) and the rectum (AAPC 7.3% [95% CI 5.6%; 9.0%]). Incident cases of annual GEP‐NEN for different sites, stratified by histologic subtype are presented in Table S1.

Over the study period, NEN incidence increased for all UICC‐stages (Table 3). The increase declined stepwise from UICC Stage I to UICC Stage IV. The strongest rise (18.7% [95% CI 13.0%; 24.8%]) was found for incidence of early stage NEN (UICC I), the lowest for NEN in an already metastasized stage (UICC IV, 3.6% [95% CI 2.2%; 5.0%]). Figure S2 shows the distribution of stages according to GEP sites.

**TABLE 3:** Trends in incidence of GEP‐NEN by UICC stage.

UICC stage	Period	ASIR 2005	ASIR 2019	AAPC (lower, upper CI 95%)	*p*‐value
UICC I	2005–2019	0.11	0.90	18.7 (13.0; 24.8)^a^	<0.001
UICC II	2005–2019	0.10	0.49	10.9 (7.8; 14.2)^a^	<0.001
UICC III	2005–2019	0.20	0.45	5.6 (2.0; 9.3)^a^	0.006
UICC IV	2005–2019	0.49	0.88	3.6 (2.2; 5.0)^a^	<0.001
UICC X	2005–2019	1.26	1.99	3.6 (2.5; 4.8)^a^	<0.001

Abbreviations: AAPC, average annual percent change; ASIR, age‐standardized incidence rate per 100,000 residents (European standard population); CI, confidence interval; GEP‐NEN, gastroenteropancreatic neuroendocrine neoplasms; UICC, Union for International Cancer Control.

^a^ Indicates that AAPC is significantly different from zero based on a significance level of 5%.

Over the study period, NEN ASIR increased for the age groups (40–59, 60–79 and 80+ years) for all sites (Figure S3). For the appendix, an increase in NEN incidence was also found for the youngest age group (0–39 years) from 0.1 to 0.8 per 100,000 residents (Figure S3). Of these, 123 were below the age of 18. Except for the appendix, incidence of GEP‐NEN increased with age.

## 4 DISCUSSION

In this analysis of population‐based data from the Bavarian Cancer Registry, an increase in ASIR of NEN was observed for most GEP sites during the study period. This increase was highest for NEN of the stomach, followed by those of the appendix, pancreas, and rectum. The increase in ASIR was observed for all stages; however, it declined stepwise from Stage I to Stage IV NEN. Rising trends of ASIR were observed for age groups 40 years and older. For NEN of the appendix, an increase of NEN incidence was additionally found in the youngest age group (0–39 years).

Continuously increasing ASIR of GEP‐NEN over the last decades were reported worldwide.^4^, ^5^, ^6^, ^16^, ^18^, ^22^ NEN incidence rates varied according to gender and ethnicity, geographic region, and tumor site.^1^, ^6^, ^22^ Few studies investigated NEN in Germany^29^ focusing either on NETs in general or on one selected GEP site.^30^, ^31^ One study from the Netherlands investigated NEN incidence trends by histologic grade using the 2010 WHO classification. Korse et al. investigated ASIR between 1990 and 2010 for the total of NEN and for different sites.^32^ To our knowledge, hitherto no study has investigated ASIR of GEP‐NEN stratified by histologic subtype according to the latest WHO classification of 2019.

The underlying reasons for the increase in GEP‐NEN incidence still remain contentious. Some studies suggest that the increase in incidence could be explained by the incidental detection of asymptomatic NEN during routine check‐up or screening.^5^, ^33^ The increasing use of advanced and more sensitive diagnostic imaging techniques in clinical practice might have enhanced detection,^4^, ^33^ which would likely lead to an increase especially of early stage tumors.^18^ Our findings support this hypothesis, as the highest increase in ASIR was found for early stage NEN (UICC I), which are characterized by a small size. Especially small and well‐differentiated rectal NEN were often found during screening colonoscopy.^33^ Similarly, we found a rise for NEN of the rectum but not the colon (excluding appendix, which usually cannot be detected by colonoscopy). In Bavaria, case numbers of screening colonoscopies increased between 2010 and 2019, simultaneously to the increase in colorectal NEN in the same period. Appendiceal NEN are often incidentally diagnosed during surgical interventions, accounting for 0.2–2.3% of appendectomies.^34^ Our data indicate that ASIR of appendiceal NEN have increased over the study period as incidence rates of appendicitis have been stable in Western countries since 1990. Appendicectomy rates even decreased.^35^ The rise in appendiceal NEN may thus be a real increase. Also, the appendix was the only GEP site, in which almost 50% of the patients were aged below 40 years. This fact is striking, as in general, the age group under 40 years is characterized by far less doctor's visits compared to persons of higher age.

Some studies conclude that the risk of developing NEN may be primarily attributable to genetic factors, however, of a hitherto unknown gene, since no correlation with the menin‐gene or any other gene is known for appendiceal NEN.^36^ A gene–environment‐interaction might also provide the explanation for the varying NEN incidence in different geographic regions.^37^ Findings of Hassan and colleagues on higher prevalence of NEN in patients whose first‐degree relatives developed any cancer^38^ suggest a possible hereditary basis. A link between NEN and cancer in general was also suggested by other studies,^39^, ^40^ supposing a shared cancer predisposition. However, the concurrence with other cancers might also be due to age, as NEN cases predominantly occur in higher age groups.^41^ Traditional risk factors such as obesity, alcohol consumption, smoking, and diabetes are assumed to increase the risk for NEN and/or GEP tumors.^41^, ^42^ Continuously rising diabetes rates^43^ and increasing trends of obese and overweight individuals^44^ could be observed for the last three decades in Bavaria and thus might have influenced the development of NEN. In contrast, a decline in prevalence rates of smokers occurred in Bavaria for the last decade and the consumption of alcohol decreased.^45^ Thus, these risk factors alone may not explain the increase of incidence in GEP‐NEN.

Our study has some limitations. Due to changes in classification, assessing trends in NETs remains challenging.^37^ We included the preceding morphology codes for the histologic GEP‐NEN subtypes and accounted for known behavior code changes, for example, the change of behavior code of the histology 8240 from 1 (“borderline malignancy”) to 3 (“malignant”) for appendiceal NEN. However, we cannot ensure that appendiceal tumors of “borderline malignancy” with the morphologic code 8240 were completely reported to the Bavarian Cancer Registry. Also, we cannot generally rule out misclassification as shifts in the classification of NEN were introduced in the recent years, especially in 2013 when the first revision of the ICD‐O3 was published.^14^ The term mixed adenoneuroendocrine carcinoma (MANEC) was introduced at first in the 2010 WHO classification of tumors of the digestive system, 4th edition.^10^ Before that date, MANEC might have been classified as NEC. The change in coding practice may have additionally contributed to the decline of reported NEC. A process of transition in registration practices is likely to have occurred prior to the introduction of the latest WHO classification of NEN in 2019,^11^ leading to the successive adoption of the novel subtype NET G3 and the decline of NEC in consequence. Moreover, until the late 90s, it used to be common clinical practice to categorize only metastasized NEN as NEC. Alterations in coding and the emergence of NEN classifications might have increased awareness of NEN in clinicians and pathologists, further enhanced by a broad implementation of improved immunohistochemical techniques.^32^ These developments may contribute to the explanation of the increase in NEN incidence.^15^ Strengths of the study are the population‐based analysis for a populous German federal state with more than 13 million residents—exceeding the populations of countries like Belgium, Sweden or Portugal–as well as the stratification by tumor site.

Analyzing a large, unselected population, this study found a significant increase in the incidence of NEN, which in the last decade was mainly driven by rising rates of NET. This increase may—at least partially—be attributable to the improved detection of tumors and changes in diagnostic classification. However, it may also be associated with changes in risk factors related to Western lifestyle and the increase of associated diseases. Further research should focus on identifying those risk factors with the highest attributable risk for GEP‐NEN to target preventive efforts accordingly and further enhance awareness towards NEN.

## AUTHOR CONTRIBUTIONS

**Nina Grundmann:** Conceptualization (equal); formal analysis (equal); investigation (equal); methodology (equal); writing – original draft (equal); writing – review and editing (equal). **Sven Voigtländer:** Conceptualization (equal); investigation (equal); methodology (equal); writing – review and editing (equal). **Amir Hakimhashemi:** Conceptualization (equal); formal analysis (equal); investigation (equal); methodology (equal); writing – review and editing (equal). **Ulrich‐Frank Pape:** Methodology (equal); writing – review and editing (equal). **Martin Meyer:** Conceptualization (equal); methodology (equal); writing – review and editing (equal). **Jacqueline Müller‐Nordhorn:** Conceptualization (equal); investigation (equal); methodology (equal); supervision (equal); writing – original draft (equal); writing – review and editing (equal).

## FUNDING INFORMATION

This research did not receive any specific grant from funding agencies in the public, commercial, or not‐for‐profit sectors.

## CONFLICT OF INTEREST STATEMENT

The authors declare no conflicts of interest.

### ETHICS STATEMENT

This observational study of routine anonymized registry data was deemed not to require formal Ethics Committee approval by the Bavarian State Chamber of Physicians (reference number: 2022‐1159).

## Supporting information


Data S1.

Table S2.

Table S3.

Table S4.

## Data Availability

The data that support the findings of this study are available in the supplementary material of this article. Additional data are available from the corresponding author upon reasonable request.
