# Supplementary material for: Site‐specific trends in gastroenteropancreatic neuroendocrine neoplasms in Bavaria, Germany
Source: Cancer Med. 2023 Sep 22;12(19):19949–58. doi: 10.1002/cam4.6510 (PMC10587981; doi:10.1002/cam4.6510)
Supplement: Supplementary file 1 — Data S1. [file CAM4-12-19949-s003.docx]

**Site-specific trends in gastroenteropancreatic neuroendocrine neoplasms in Bavaria, Germany**

Nina Grundmann^1^, Sven Voigtländer^1^, Amir Hakimhashemi^1^, Ulrich-Frank Pape^2^, Martin Meyer^1^, Jacqueline Müller-Nordhorn^1^

^1^ Bavarian Cancer Registry, Bavarian Health and Food Safety Authority, Nuremberg, Germany

² Department of Internal Medicine and Gastroenterology, Asklepios Tumour Centre Hamburg and Asklepios Hospital St. Georg, Hamburg, Germany

**Supplementary data**

**Supplementary Table 1. Trends in GEP-NEN incidence by site**

| **Site** | **Period** | **ASIR 2005** | **ASIR 2019** | **AAPC (lower,  upper CI 95%)** | **p-value** |
| --- | --- | --- | --- | --- | --- |
| GEP-NEN (all) | 2005-2019 | 2.18 | 4.76 | 5.9 (4.5; 7.2)* | <0.001 |
| Oesophagus | 2005-2019 | 0.02 | 0.06 | 6.9 (3.3; 10.8)* | 0.001 |
| Stomach | 2005-2019 | 0.26 | 0.57 | 8.3 (6.4; 10.2)* | <0.001 |
| Small intestine | 2005-2019 | 0.77 | 1.09 | 3.3 (2.1; 4.6)* | <0.001 |
| Colon | 2005-2019 | 0.13 | 0.30 | 1.9 (-0.7; 4.5) | 0.140 |
| Appendix | 2005-2019 | 0.27 | 0.77 | 8.1 (4.4; 12.0)* | <0.001 |
| Rectum | 2005-2019 | 0.27 | 0.63 | 7.3 (5.6; 9.0)* | <0.001 |
| Bile/ Liver | 2005-2019 | 0.04 | 0.12 | 3.6 (0.4; 6.9)* | <0.031 |
| Pancreas | 2005-2019 | 0.42 | 1.21 | 7.7 (5. 6; 9.7)* | <0.001 |

Abbreviations: AAPC: average annual percent change, ASIR: age-standardized incidence rate per 100,000 residents (European standard population) CI: confidence interval, GEP-NEN: gastroenteropancreatic neuroendocrine neoplasms.
* indicates that AAPC is significantly different from zero based on a significance level of 5%.

**
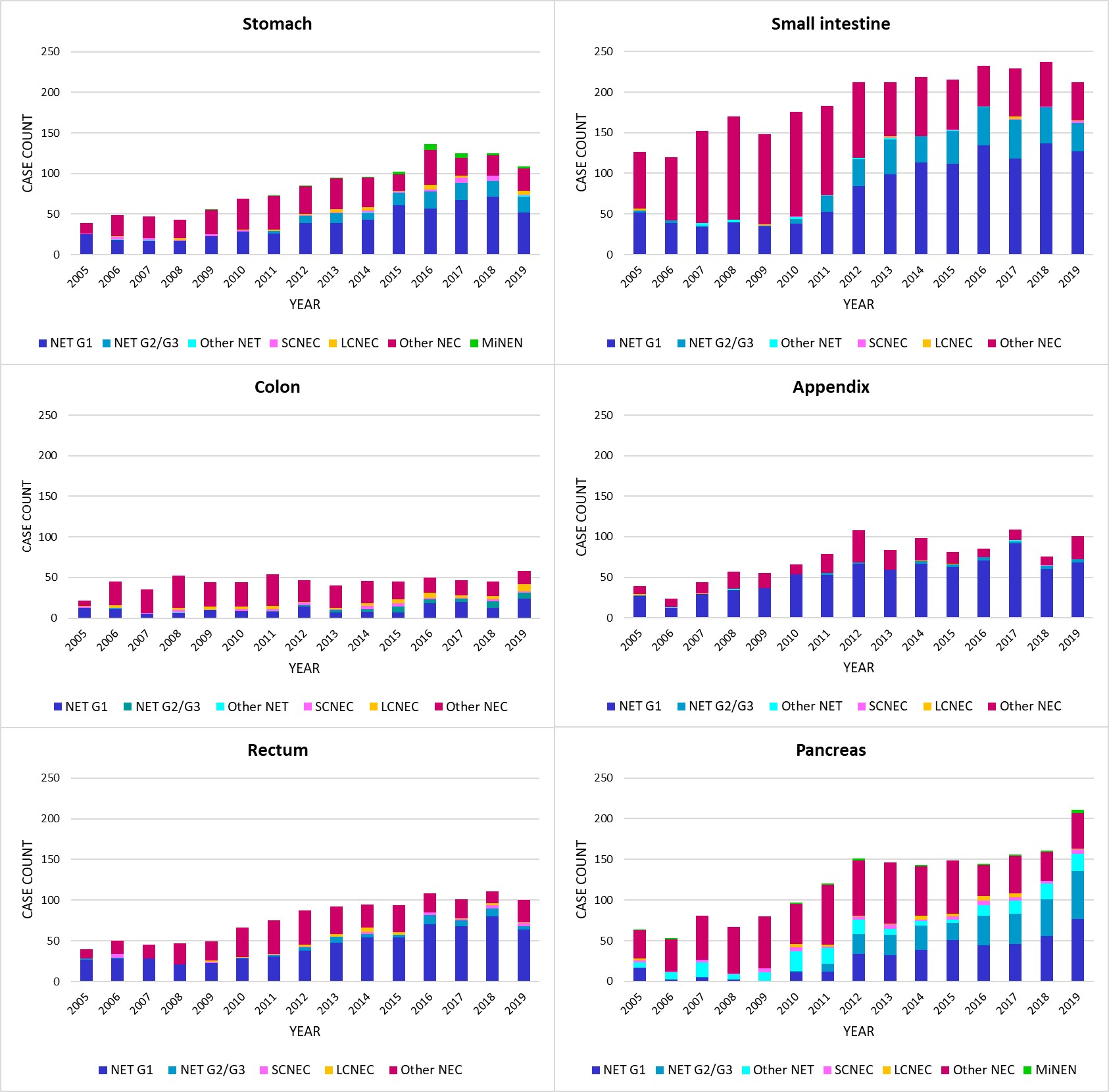
**

**Supplementary Figure 1:** **Incident cases of GEP-NEN for different sites, stratified by histologic subtype**
Abbreviations: GEP-NEN: gastroenteropancreatic neuroendocrine neoplasm, G: grade, MiNEN: mixed neuroendocrine-non-neuroendocrine neoplasm, NEC: neuroendocrine carcinoma, NET: neuroendocrine tumor, LC: large cell, SC: small cell

**
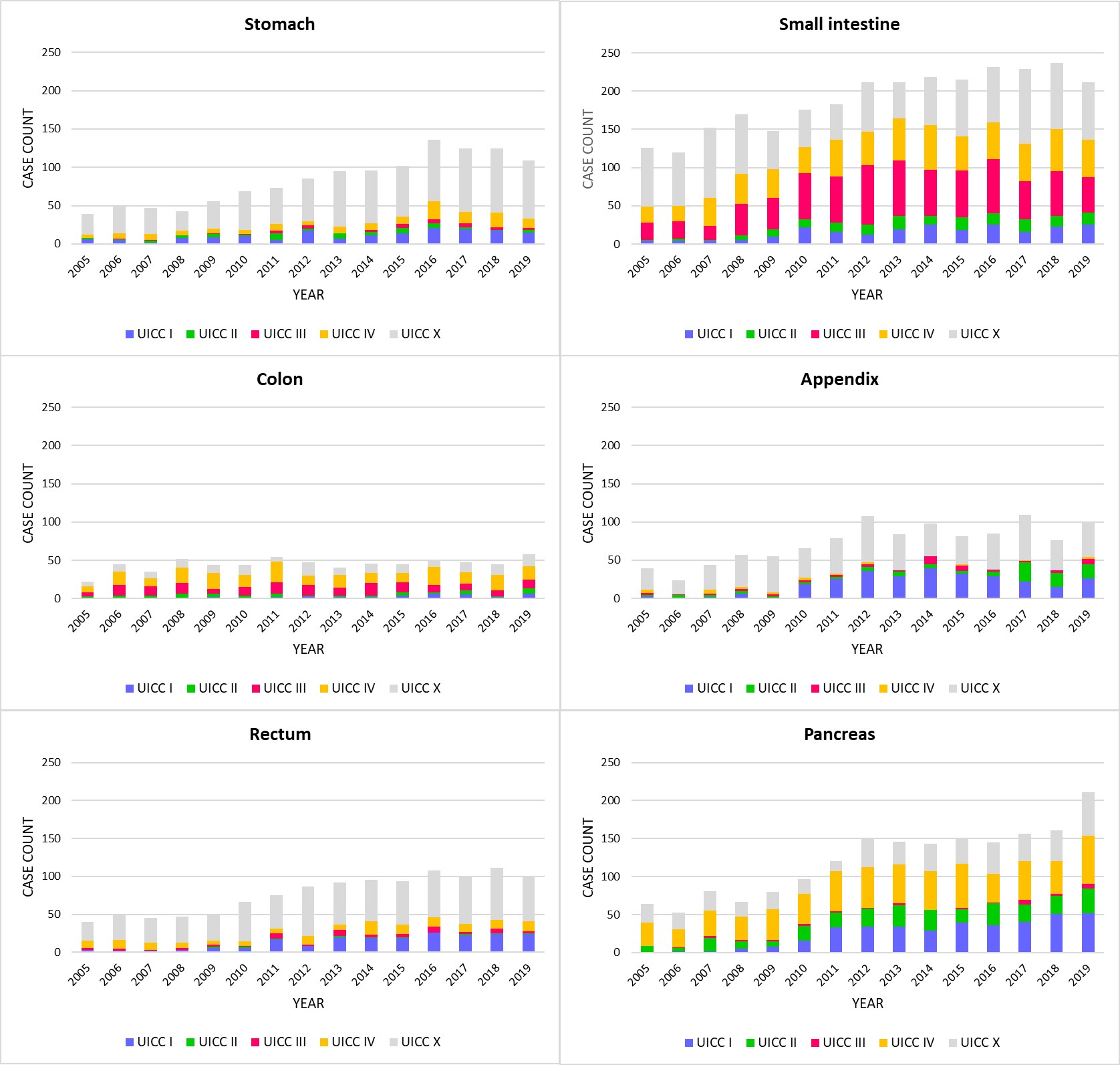
**

**Supplementary Figure 2:** **Incident cases of GEP-NEN for different sites, stratified by UICC-stage**
Abbreviations: GEP-NEN: gastroenteropancreatic neuroendocrine neoplasm, UICC: Union for International Cancer Control


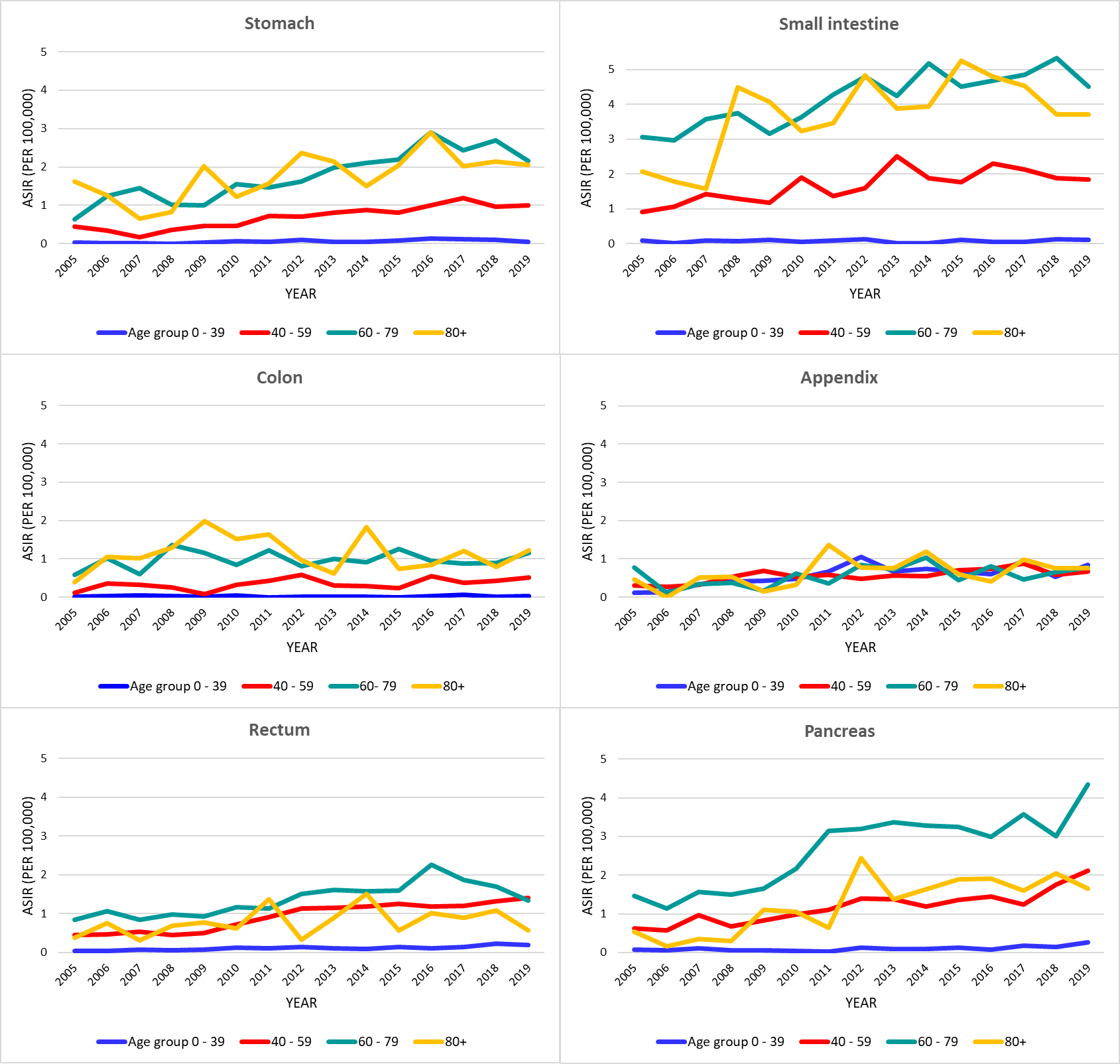


**Supplementary Figure 3:** **Age-standardized incidence for GEP-NEN of different sites, stratified by age group**Abbreviations: ASIR: age-standardized incidence rate (European standard population), GEP-NEN: gastroenteropancreatic neuroendocrine neoplasm
